# Urinary Lipocalin Protein in a Female Rodent with Correlation to Phases in the Estrous Cycle: An Experimental Study Accompanied by *In Silico* Analysis

**DOI:** 10.1371/journal.pone.0071357

**Published:** 2013-08-14

**Authors:** Subramanian Muthukumar, Durairaj Rajesh, Ganesan Saibaba, Alagersamy Alagesan, Rengasamy Lakhsminarayanan Rengarajan, Govindaraju Archunan

**Affiliations:** 1 Centre for Pheromone Technology, Department of Animal Science, School of Life Sciences, Bharathidasan University, Tiruchirappalli, India; 2 The Swire Institute of Marine Science and School of Biological Sciences, The University of Hong Kong, Hong Kong, SAR; Duke University, United States of America

## Abstract

Male urinary lipocalin family proteins, practically odorant-binding proteins but also could be pheromones by themselves, in rodents act as a shuttle for chemosignal communication and facilitate delivery of the signals for access to congeners. However, presence of this protein in urine of female rodents has not yet been reported. Therefore, the present investigation was carried out to find if lipocalin family protein is present in the urine of female house rat and, if so, to find whether its expression differs between the phases in the estrous cycle. The rat urinary protein was separated in single dimensional gel electrophoresis. A 14.5 kDa lipocalin protein appeared in the urine prominently during the estrus and metestrus phases compared to proestrus and diestrus phases. The expression of this protein in the urine was very low in ovariectomized rats. MALDI-TOF/MS analysis affirmed the 14.5 kDa protein as a lipocalin family protein. Analysis adopting bio-informatics tools further proved the protein as a lipocalin family member. Thus, this study for the first time demonstrated the presence of a lipocalin family protein in the urine of a female rodent and it was highly expressed during estrus phase. This lipocalin protein in female rat urine may facilitate a chemosignal function independently of a pheromone or in association with a specific pheromone.

## Introduction

Chemosignals are small volatile compounds associated in one or more ways in synchronization of aspects of reproductive physiology between males and females and mediation of social responses [1], [2]. Rodents excrete large amounts of proteins in their urine which play a significant role in the delivery of chemical messages [3], [4]. The volatile urinary compounds of mouse bind with carrier proteins called major urinary proteins (MUPs) [3]. A comparable functional protein, α-2u globulin, has been identified in the rat [5]. Both MUPs and α-2u globulin belong to the structural homology superfamily called lipocalin [6], [7]. A variety of small volatile molecules present in the male mouse urine (brevicomin, thiazole) bind with the MUPs and produce wide-ranging effects in the conspecifics [8], [9], [10]. Furthermore, it has been demonstrated that MUP-pheromone binding in male mice urine provides for a gradual release of the volatile ligand to the environment [11] thereby enhancing the longevity of the volatile. This finding provides strong circumstantial evidence that the lipocalin (MUP) is capable of releasing the volatiles at a slow pace, suggesting a lead to a biological application. Similarly, in the urine of male rat a volatile was found in a form bound to α-2u globulin [12], and pheromone-α-2u globulin binding was also traced to the preputial gland which is one of the major sources of volatiles [5]. Thus, the lipocalin proteins present in the male possess the ability to bind and release the volatiles and participate in several pheromone-based effects in the conspecifics.

Looking for a role for the urinary lipocalin proteins in male rodents, it has been shown that the proteins by themselves might act as pheromones or associate with specific volatiles so as to release them in small quanta and bring about pheromonal effects on the conspecifics. The MUP, along with its bound volatile, has been found to be involved in several primer pheromonal effects including male-induced pregnancy block (Bruce effect) and acceleration of puberty (Vandenbergh effect) in female mice [13], [14]. Aphrodisin, a lipocalin present in hamster vaginal discharge, acts as a reproductive pheromone [15]. A low molecular weight involatile protein darcin, identified in male mouse urine, has been found to act as a male sex pheromone and attract female mice [16].

Analysis of the chemosignal-binding protein at the levels of primary and tertiary structures in relation to the lipocalin family proteins strongly suggested that the chemosignal-binding proteins belong to the lipocalin family [17]. The tertiary structure of MUPs expounds hydrophobic residues which would provide for ligand-binding ability and protein stability [18]. The 2.4 Å crystal structure, a conserved β-barrel structure, in MUP-I isolated from mouse urine [6] indicates that MUPs are members of the lipocalin family protein [19]. Crystal structures have been determined for several other lipocalins, including serum retinol-binding protein [20], β-lactoglobulin [21], bilin-binding protein [22], and α-2u globulin [6].

During estrous cycle, the females differ in physiological status and hormonal regulation, which would influence their chemical cues for the attraction towards the partner. During estrus, a specific volatile signal would pass from female to male partner through urine as a mating call, in which the lipocalin protein may play role as a chemosignal shuttle [23]. Estrus-specific chemosignals have been reported in the urine of rat [24], mouse [25], cow [26], [27], buffalo [28], [29] and elephant [30]. However, even though there are well documented studies on lipocalin protein in the urine of male rodents, there is no specific report on presence of lipocalin proteins in urine of female rodent. Therefore, we undertook this study to find the presence of lipocalin protein in commensal rat urine and to perceive their expressional difference with reference to the estrous cycle.

## Materials and Methods

### Ethics Statement

The procedures used in the present study were approved by the Institutional Animal Ethics Committee (IAEC) of Bharathidasan University, India, under the regulatory control of Committee for the Purpose of Control Supervision on Experiments on Animals (CPCSEA), Government of India (Approval No. BDU/IAEC/2012/61) and the rats were maintained at the animal house facility, Department of Animal Science, Bharathidasan University, Tiruchirappalli, India.

### Experimental Animals

The rat species used in the present study, commensal rat (*Rattus rattus*), is not included in the list of endangered/protected animals. The rats were trapped at residential areas close to Bharathidasan University, with proper permission from the house owners. All animals thus collected were transferred to the animal house maintained by Department of Animal Science, Bharathidasan University, India. Reproductively active female rats weighing ∼150–200 g were selected and allowed to acclimate to laboratory conditions for one month. After laboratory acclimation, animals with regular estrous cycle were selected for further experimental study. The rats were housed in polypropylene cages (40×25×15) with 2 cm of rice husk lining the bottom as bedding material. The bedding material was changed once every three days to maintain the hygienic condition. All animals were maintained in 12∶12 L & D cycle throughout the experimental period.

### Determination of Phases of Estrous Cycle

For identification of the phases of estrous cycle, the vaginal smear was analyzed under light microscope and find out the proportion of three cell types namely nucleated epithelial cells, cornified epithelial cells and leucocytes [31], [32]. Proestrus was indicated when nucleated epithelial cells alone were present; estrus when cornified epithelial cells alone were present; metestrus when both cornified epithelial cells and leucocytes were present; and diestrus when leucocytes alone were present. This assessment was done before and after every urine sample collection.

### Vaginal Cytology by SEM (Scanning Electron Microscopy) Analysis

Based on the cellular constituents in the vaginal smear during estrus and diestrus phases, the cells were subjected to scanning electron microscopic analysis. A cold Field Emission–Scanning Electron Microscope (FE–SEM, 6701 F, JEOL, Japan) at the Center for Nanotechnology and Advanced Biomaterials (CeNTAB), SASTRA University, Thanjavur, India, was used. The spot of smear sample was spread in a glass slide and mounted on to a brass stub using a double sided carbon tape. The samples surface was sputter coated with gold particle in a thin film at a current of 20 mA for 45 sec to form a conducting layer. A brass stub was loaded into the specimen chamber. An accelerating voltage of 3 kV was used to image the samples at ultra-high vacuum using the secondary electron detector and working distance of 6.5 to 7 mm [33]. The cells were photographed.

### Urine Collection and Sample Preparation

Urine from females rats (n = 12), which exhibited regular estrous cycle, was collected for over ten consecutive estrous cycles. In order to avoid the contaminants such as hair, dirt and fecal debris, the animals were shifted to specialized metabolism cages (without bedding) for urine collection. The collected samples were immediately filtered though Whatman filter paper and centrifuged at 12,000 rpm for 10 min at 4°C, and the clear supernatant was separated. The samples were concentrated using 10 kDa Centricon cut-off membrane (GE Biosciences). The concentration of protein was determined according to Bradford [34].

### Ovariectomy

Reproductively active female rats (n = 5) were anesthetized using diethyl ether. The abdominal region was shaved and wiped with ethanol. A single midline incision in lower abdominal region was made using a surgical blade. The oviducts were pulled out and both ovaries were removed. Afterwards the inner and outer skin layers were stitched [35]. Three weeks later, the vaginal cytology was observed for loss of cyclicity. Urine was collected from the ovariectomized rats to find the expression of the protein of interest.

### Sodium Dodecyl Sulphate Poly Acrylamide Gel Electrophoresis (SDS-PAGE)

In the present investigation, 12% SDS-PAGE was carried out with female rat urinary protein from each phase of the estrous cycle, and from ovariectomized rat (30 µg protein in each). A medium range molecular weight marker set (Bangalore Genei) was used for the molecular weight reference. The electrophoresis was done at constant voltage (50 V) at room temperature.

### Detection of Protein by Coomassie Brilliant Blue Solution

After completing the electrophoresis, the gels were rinsed with distilled water for 2 min and stained with 0.5% Coomassie brilliant blue R-250 in 40% methanol and 10% acetic acid, at room temperature, for 2 hrs. The gels were then destained in a solution containing 40% methanol and 10% acetic acid until background became clear. Finally, the gels were washed with Milli-Q water and utilized for further analysis. The intensity and molecular weight of the protein bands in the gels were identified using gel documentation (Quantity One software, Bio Rad, CA, USA). The band area was measured in pixels.

### Trypsin Digestion

The trypsin in-gel digestion of urinary polypeptides was conducted according to Rajkumar et al. [5]. The differentially expressed bands were removed from the SDS-PAGE using a thin glass pipette and placed into microcentrifuge tubes. The gel plug was destained using 100 µL of 50 mM ammonium bicarbonate and 50% (v/v) acetonitrile (1∶1) and incubated at 37°C for 30 min. This step was repeated until no stain was visible in the gel spot. Gel pieces were sliced into small cubes, and placed in 1.5 mL tube pre washed with ethanol.

The plugs were then incubated at 37°C with 50 µL of 10 mM DTT for 30 min; the DTT was discarded, and 50 µL of 55 mM iodoacetamide was added to each tube and incubated for 1 hr at room temperature in the dark. The iodoacetamide solution was discarded and the plugs were washed twice as above before being dehydrated in 100% acetonitrile and then rehydrated in 9 µL of 50 mM ammonium bicarbonate. After 30 min incubation at room temperature in the dark, the gel particles were washed with 50% acetonitrile in 0.1 M ammonium bicarbonate and dried in a Speed-Vac evaporator [36]. The dried gel pieces were swollen in a minimum volume of a 10 µL digestion buffer that contained 50 mM ammonium bicarbonate, 5 mM calcium chloride, and 100 ng of trypsin in order to keep the gel pieces wet during enzymatic cleavage at 37°C for overnight.

### MALDI-TOF/Mass Spectrometry

The sample (tryptic fragmented) was prepared by mixing equal amounts (2∶2) of peptide mixture with the matrix solution (α-Cyano-4-hydroxycinnamic acid) saturated with 0.1% TFA and acetonitrile (1∶1). Then the samples were analyzed in *Ultraflex* spectrometer (Brukar Daltonics, Billericia, MA, USA and Bremen, Germany) with reflection mode a delay time of 90 ns and 25 kV accelerating voltage in the positive ion mode. To improve the signal to noise ratio summation of 300 laser shots were taken for each spectrum. External calibration was done using peptide I calibration standard with masses ranging from 1046–3147 Da. Mass spectra were acquired by selecting the precursor mass with 8 Da window.

### Database Analysis

The collected mass spectra were processed by FLEX analysis software and the mono- isotopic peptide masses were assigned and used in the database search. The protein identification was accomplished using MASCOT search (http//www.matrixscience.com). Scores >63 were considered to be significant (p<0.05). The primary sequence detected in female rat urinary protein was retrieved from NCBI. The amino acid composition and physiochemical properties viz., molecular weight, theoretical pI, amino acid length, atomic composition, estimated half life, instability index, extinction coefficient, aliphatic index, and grand average of hydropathicity (GRAVY) were computed by PROTPARAM tool available in EXPASY website (http://web.expasy.org/protparam/). Further analysis for finding domain region and motif in the primary sequence was done by DIAL server (http://caps.ncbs.res.in/DIAL/home.html) [37]. Prediction of helix, sheets and coils in the secondary structure of the lipocalin was carried out using Self-optimized prediction method with multiple alignments (SOPMA) (http://npsa-pbil.ibcp.fr/cgi-bin/npsa_automat.pl?page=/NPSA/npsa_sopma.html) [38].

### Molecular Modeling and Structural Validation

The lipocalin sequence was compared with the other urinary lipocalin proteins and fatty acid binding protein collected from non-redundant database i.e., NCBI (www.ncbi.nlm.nih.gov) through multiple sequence alignment (MSA) executed by ClustalW2 (http://www.ebi.ac.uk/Tools/msa/clustalw2/), which is available in EMBL-EBI website and Expasy bioinformatics resource portal [39].

Comparative modeling of lipocalin was done for determining the three dimensional structure and binding sites. SWISS-MODEL (http://swissmodel.expasy.org/) [40] is a database for constructing the homology model of protein or DNA with computational approach to predict the best hit template for the unknown or desired sequence. The lipocalin family protein was used to identify the best template using template identification tool in the SWISS-MODEL database. Molecular modeling creates an accurate model of the lipocalin sequence by comparing the best hit template. The (PS)^ 2^-V2 is a molecular modeling server that consists of MODELLER package was used to constructing three dimensional structure of lipocalin protein through automated method (http://ps2v2.life.nctu.edu.tw/) [41].

Model validation carried out by SAVeS server, in which PROCHECK tool was selected (http://www.ebi.ac.uk/thornton-srv/software/PROCHECK/) [42]. Computationally calculated phi/psi values and allowed and disallowed regions of desired protein model were made using Ramachandran plot, which is mainly performed for predicting range of helices, strands and coils of furnished model. Hence, the visualization of the constructed model was performed using PyMol software (V0.99) (http://www.pymol.org/). The binding sites were predicted using Q-SiteFinder server (http://www.modelling.leeds.ac.uk/qsitefinder/) [43].

### Statistics

The band intensity of estrous cycle and ovariectomized animal urinary protein was analyzed using one-way analysis of variance (ANOVA) by SPSS version 16 (SPSS Inc., Cary, NC, USA).

## Results and Discussion

### Characterization of Vaginal Smear and SEM Analysis

**Figure 1:**
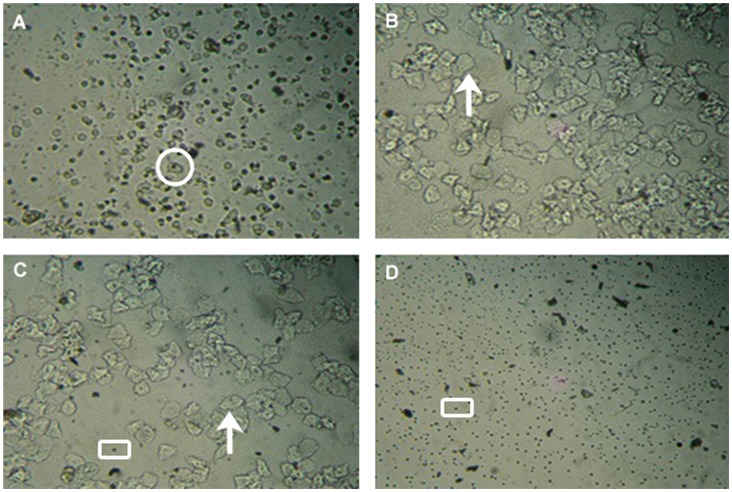
Vaginal cytology as observed in commensal rat at different phases of estrous cycle. The observed estrous cycle phases are (A) Proestrus, (B) Estrus, (C) Metestrus, (D) Diestrus. Nucleated epithelial (circle), cornified epithelial (arrow), leucocytes (Square) were identified in vaginal smear.

Proestrus was identified from the presence, in vaginal smear, of round, nucleated epithelial cells with granular structures (Figure 1A). The first onset of this feature identified day one of the estrous cycle. The proestrus most invariably commenced at early morning, from 5 to 8 am, and it was never delayed beyond the evening of day of first appearance. During transition to estrus the vaginal smear showed a characteristic cluster of cornified epithelial cells. At the end of the estrus, the cells changed in appearance to needle-like due to losing of content. In as much as one estrous cycle covered five days, estrus lasted mostly for only one day, and very occasionally two days (Figure 1B). Metestrus preceded diestrus and during this time the smear had fewer cornified epithelial cells but mostly leucocytes. This transition phase lasted only few hours of the entire cycle (Figure 1C). When the smear showed exclusively leucocytes populations the day was considered as diestrus. This condition continued for two or three days in each cycle (Figure 1D). By the close of diestrus, in addition to the leucocytes, a few epithelial cells appeared in the smear. SEM analysis revealed the presence of cornified cells during estrus and leucocytes during diestrus (Figure S1).

### SDS-PAGE of Rat Urinary Protein

**Figure 2:**
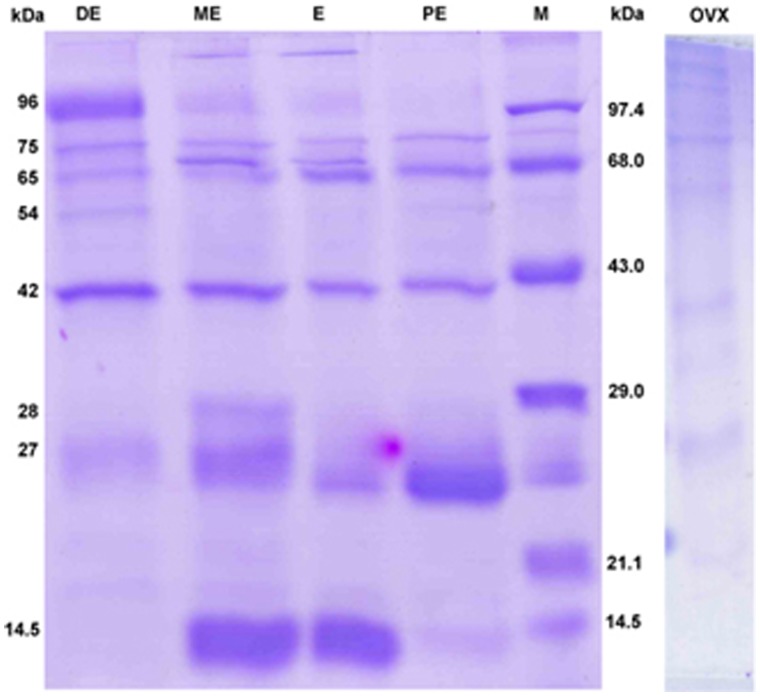
SDS-PAGE urinary protein profile of commensal rat. The estrous cyclic and ovariectomized urinary proteins were separated by 12% SDS-PAGE and the Lane- M contains 7 µL of Bangalore Genei protein molecular markers, (PE) Proestrus, (E) Estrus, (ME) Metestrus, (DE) Diestrus, (OVX) Ovariectomized, each lane contains 30 µg protein.

**Figure 3:**
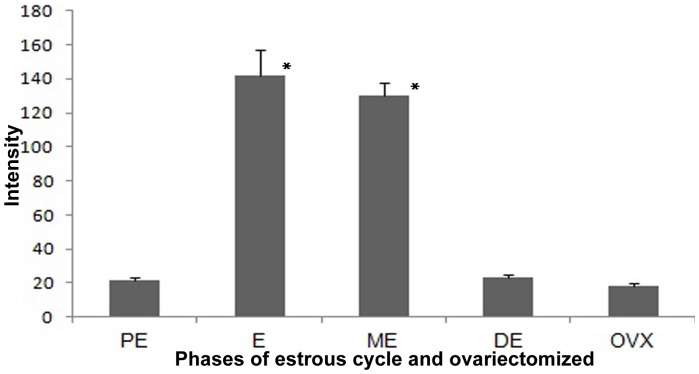
Intensity (band area) of 14.5 kDa protein around estrous cycle and Ovariectomized animal. The intensity of the protein band was compared with estrous phases and ovariectomized animal. The protein intensity significantly high during estrus (E) and metestrus (ME) compared to proestrus (PE), diestrus (DE) and ovariectomized (OVX) using Fisher’s least significant difference post-hoc comparisons (*p<0.05). Values are mean ± SE from six gels (Figure S2).

The urinary protein profiles of rat during the four phases of estrous cycle were compared. Irrespective of the phases in the estrous cycle, totally eight polypeptides were expounded by Coomassie brilliant blue staining of the gels. The molecular mass of these polypeptides ranged from 14.5 to 96 kDa (Figures 2 and S2). The molecular weights were identified through medium range molecular weight markers. The polypeptides of high molecular weight did not differ much between phases. On the other hand the intensity of low molecular weight polypeptides differed between the phases. The 28 kDa polypeptide appeared only during metestrus. The 27 kDa protein was highly expressed during proestrus, and gradually decreased through the subsequent phases. It is known that pheromonal communications are mediated by lipocalin proteins of molecular mass, from 17 to 30 kDa [9], [12], [44]. In the present study, 14.5 kDa protein appeared faintly during proestrus, reaching the highest intensity during estrus and metestrus (p<0.05) and then it almost disappeared during diestrus. However, in ovariectomized animals 14.5 kDa protein was almost absent (Figures 2, 3, and S2).

Recently, Roberts et al. [16] have reported that 18.89 kDa involatile protein in male mouse urine, darcin, acts as a sex attractant. Darcin is also actively involved in spatial learning of female mice for individual identification lasting 14 days [45]. This urinary protein, proved to be involved as signaling pheromone, is first report in rodent chemical communication. It is interesting that a 14.5 kDa protein was identified in the present study in female rat urine, and it was expressed at a significantly higher intensity during estrus and metestrus. It is to be remembered that metestrus is only a transient extension of estrus and is short-lived. Moreover, this protein is expressed poorly in ovariectomized rat urine. Hence, the results convincingly suggest that this protein may signify something special during estrus and may have a role in conspecific communication through pheromonal compounds or it may act as a sex attractant by itself.

### MALDI-TOF/MS

**Figure 4:**
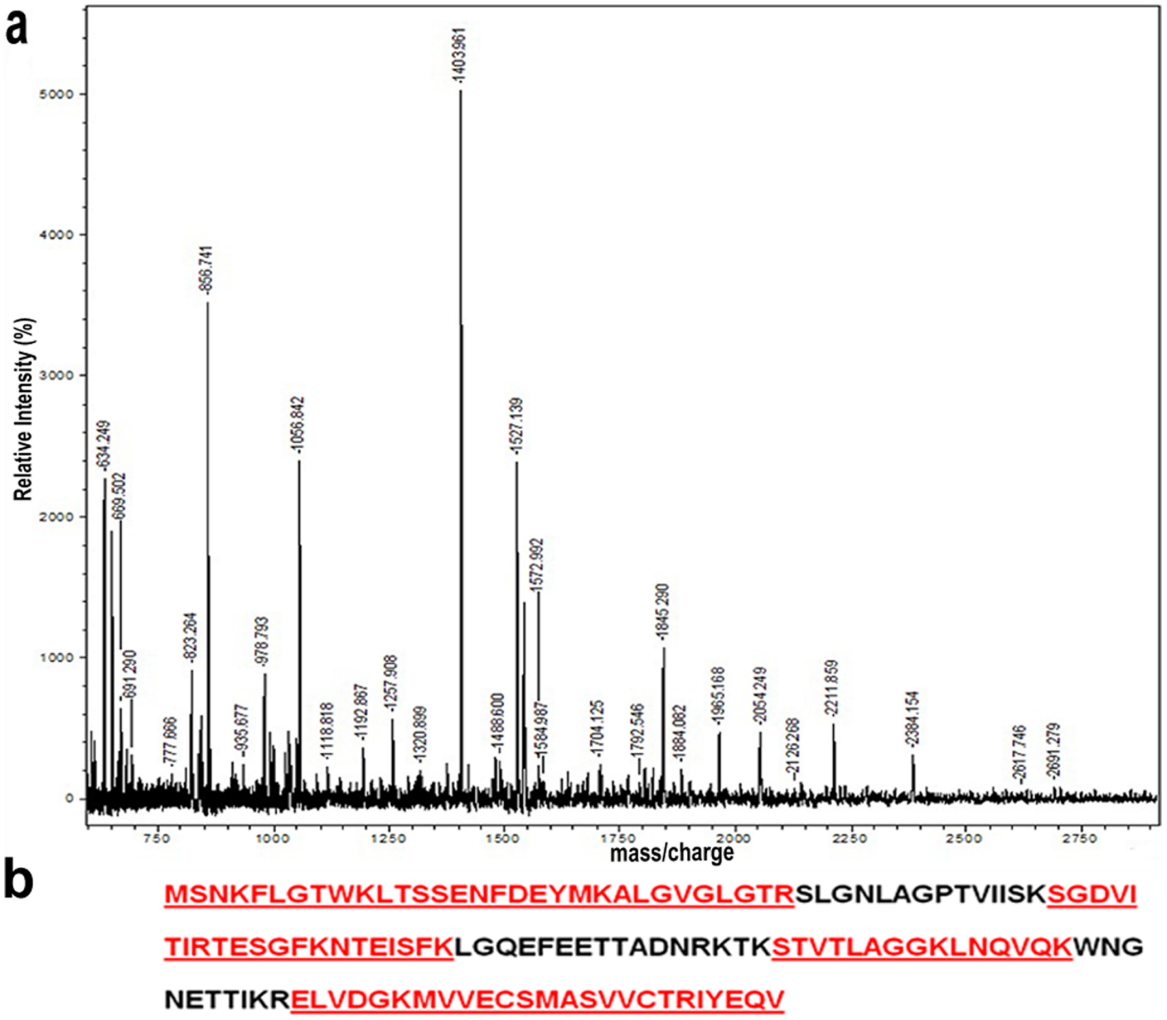
MALDI-mass spectrum and Sequence coverage of 14.5 kDa band. (a) 14.5 kDa protein band was undergone for in-gel tryptic digestion and the spectra was collected form MALDI-MS. Number in the mass spectrum gives precise m/z (M+H) values for detected peptide ion signals. (b) Single letter coded protein sequence was obtained for 14.5 kDa from mascot search. The matched 69% sequence coverage was highlighted in underlined red colour.

The differentially expressed 14.5, 27, 28 and 96 kDa proteins were excised and subjected to in gel-tryptic digestion followed by peptide mass finger printing (PMF). Good quality MALDI spectra were obtained for 14.5 kDa band (Figure 4a). Polypeptides of MW 27, 28, 96 kDa did not match with any lipocalin family protein (data not included). Further, the monoisotopic mass of 14.5 kDa protein was subjected to MASCOT search to identify the full length sequence. It closely matched with *Equus caballus* lipocalin family protein with 69% sequence coverage and three matched peptides (Figure 4b, Table 1). Here-in-after this protein is referred to as estrus urinary lipocalin protein (EULP) of rat. This result is consistent with the earlier reports on lipocalin protein and EULP may be considered as the vehicle for communication through/as a volatile compound from delivery to perception [5], [9], [46].

**Table 1:** Mass values of EULP.

Start - End	Observed Mass	Expected Mass	Sequence
**1–10**	1211.0410	1210.0337	-.MSNKFLGTWK.L
**5–22**	2211.8590	2210.8517	**K.FLGTWKLTSSENFDEYMK.A**
**23–31**	842.7690	841.7617	K.ALGVGLGTR.S
**46–53**	861.2150	860.2077	K.SGDVITIR.T
**54–59**	669.5020	668.4947	**R.TESGFK.N**
**54–66**	1488.6000	1487.5927	**R.TESGFKNTEISFK.L**
**83–97**	1543.0430	1542.0357	K.STVTLAGGKLNQVQK.W
**108–113**	660.6750	659.6677	R.ELVDGK.M
**114–127**	1660.1350	1639.1277	K.MVVECSMASVVCTR.T
**128–132**	650.2190	649.2117	R.IYEQV.-

Observed and expected masses (M+H) of 14.5 kDa protein and tryptic digested peptide sequence map by MALDI-TOF/MS, which was retrieved from Mascot database. The matched peptides are bolded in black.

### Amino Acid Properties of EULP

The physicochemical properties of EULP were analyzed using the PROTPARAM tool in Expasy bioinformatics resource portal. The EULP showed up to about 132 amino acids, and the total number of atoms in the sequence was 2043. The sequence purified from the lowest molecular weight at 14,491.5 and the theoretical pI was 8.56. The sequence formula was C_632_H_1029_N_173_O_203_S_6._ The EULP sequence contains about 15 positively charged residues (Arg and Lys combined) and 15 negatively charged residues (Asp and Glu combined). The aliphatic index was calculated as 78.18. The instability index of the protein was computed as 32.20 and stable. The grand average of hydropathicity (GRAVY) was calculated as -0.326, and it is indicative of hydrophilic and soluble protein. Proline showed single residue in minimum number (0.80%), and the single largest number of amino acid present in the sequence was threonine (11.40%). However, histidine (0.00%), pyrrolysine (0.00%) and selenocysteine (0.00%) were totally absent (Table 2).

**Table 2:** EULP sequence information.

Amino acid	No of residues	Percentage of residues
Ala (A)	5	3.80
Arg (R)	5	3.80
Asn (N)	8	6.10
Asp (D)	4	3.00
Cys (C)	2	1.50
Gln (Q)	4	3.00
Glu (E)	11	8.30
Gly (G)	13	9.80
His (H)	0	0.00
Ile (I)	7	5.30
Leu (L)	10	7.60
Lys (K)	12	9.10
Met (M)	4	3.00
Phe (F)	5	3.80
Pro (P)	1	0.80
Ser (S)	11	8.30
Thr (T)	15	11.40
Trp (W)	2	1.50
Tyr (Y)	2	1.50
Val (V)	11	8.30
Pyl (O)	0	0.00
Sec (U)	0	0.00

EULP amino acid sequence information was retrieved from the PROTPARAM tool in EXPASY bioinformatics resource portal.

### Domain Identification by DIAL Server

The domain is an important element of protein, which can provide for activation of the structural and functional expression in a cell. The EULP sequence was submitted for the identification of domain present in the entire sequence. Six significant functional motifs were identified in EULP sequence; they are an N-glycosylation site, a tyrosine sulfation site, seven protein kinase C phosphorylation site, a casein kinase II phosphorylation site, two N-myristoylation site, and a cytosolic fatty-acid binding protein site [37] (Table S1).

### Secondary Structure Prediction by SOPMA

**Figure 5:**
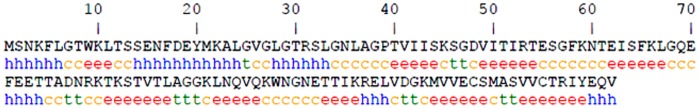
Secondary structure of EULP. Secondary structure of 14.5 kDa protein was analyzed by SOPMA tool. The total sequence length was 132 and the sequence contains extended strand (Ee), alpha helix (Hh), random coil (Cc) and beta turn (Tt).

SOPMA is fully based on the homologue method as proposed by NPS@ (Network Protein Sequence Analysis) web server. EULP sequence having extended strand (Ee) was found most frequent (37.12%), followed by random coil (Cc) that was 28.79% and alpha helix (Hh) that was 25.00%. The beta turn (Tt) was found to least frequent, 9.09% (Figure 5).

### Multiple Sequence Alignment by ClustalW2

**Figure 6:**
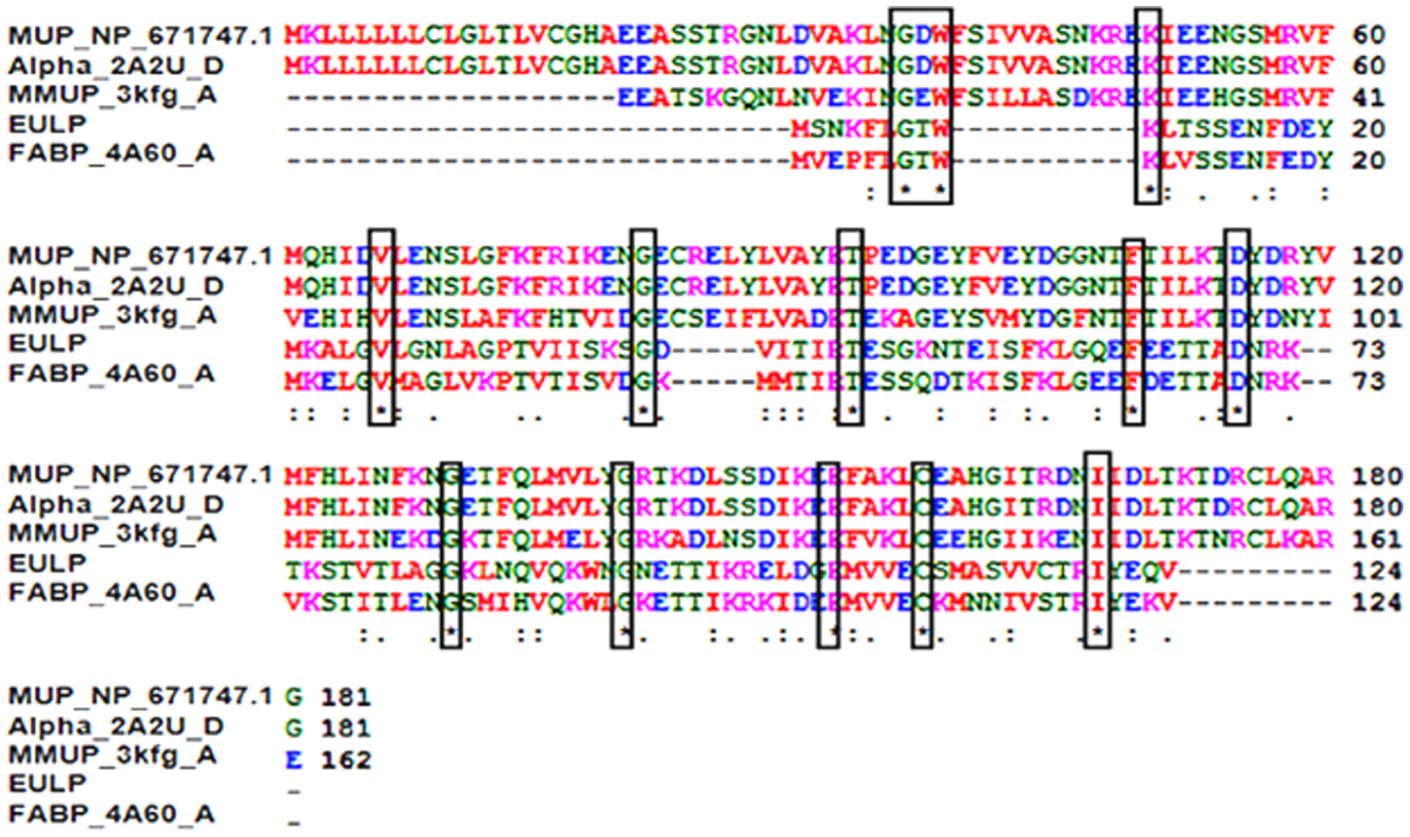
MSA analysis of EULP with other lipocalin proteins. MSA alignment made by using major urinary protein (Gene ID: NP_671747.1) followed by a-2u globulin (PDB code: 2A2U), major mouse urinary protein (PDB code: 3KFG), fatty acid binding protein (PDB code: 4A60) and EULP. EULP have 12 conserved motif (*) denoted in the boxes, -GXW- motif region and many identical residue (**:**).

The EULP sequence were aligned with MMUP (Mouse major urinary protein_3kfg_A), MUP (Major urinary protein_NP_671747.1), α2u globulin (A2u_2A2U_D) and a fatty acid binding protein (FABP_4A60_A), respectively, and showed the maximum numbers of identical residues and also represented the most frequent numbers of conserved residues (Figure 6) [12]. Lipocalin members have some specific motifs within the family [19]. Most probably, the sequence of EULP has a significant lipocalin motif (i.e. -GXW- region) with the other urinary protein of rodents and fatty acid binding protein. Hence, the -GXW- is the major reported feature of lipocalin protein [47] and significantly present in the EULP. This is the ultimate proof that the EULP fulfills the requirements to be identified as a lipocalin super family protein, and it may be associated with the delivery of a chemosignal during the estrus phase.

### Homology Modeling and Structure Validation

Most similar sequences were analyzed and the highest hit was used as a template for the EULP made by SWISS-MODEL database. The first hit from the HH search was human testis-specific fatty acid binding protein (PDB code: 4A60_HTSFABP). The EULP and template were matched with highest probability, the aligned residues are 131, E-value is 9.8e-45, the sequence identity is 63% and alignment score is 292.88.

**Figure 7:**
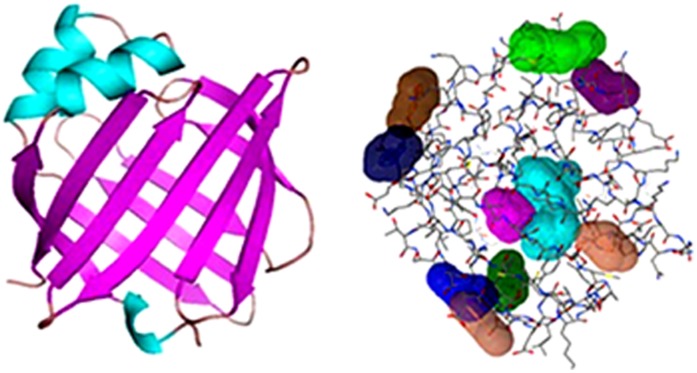
Homology modeling and binding site prediction for EULP. (a) EULP secondary structure was model by (PS)^2^-V2 and visualized in PyMol. The model represents the alpha helices and beta sheets were linked with coils and look like TIM Barrel structure. (b) Binding sites was analyzed by Q-SiteFinder and the protein expresses 10 different binding sites (different colours).

**Figure 8:**
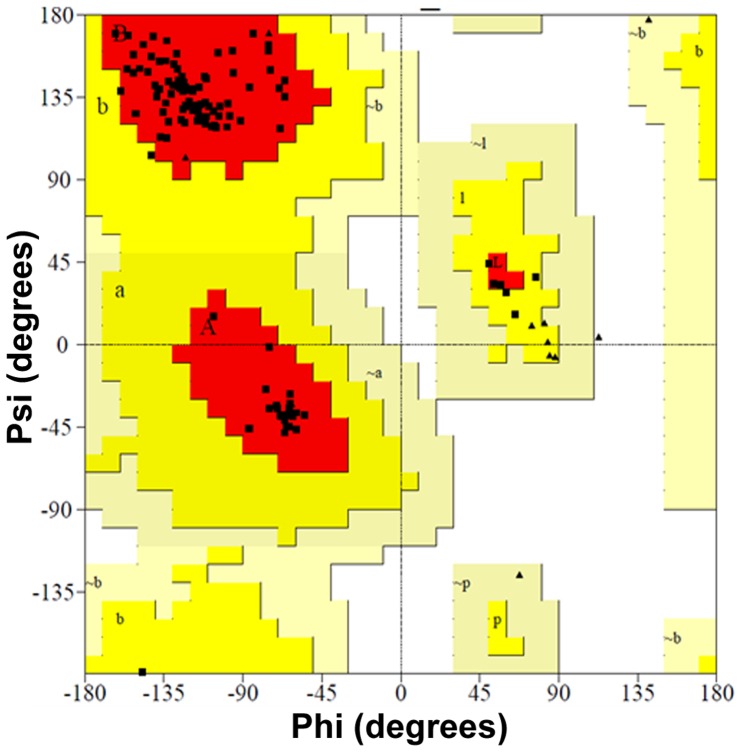
Structure validation of EULP. The developed EULP model was validated by Ramachandran Plot using PROCHECK tool in SAVeS server. The result showed that 95.7% residues were present in most favoured regions and 4.3% residues in the additional allowed regions.

The EULP model was created using protein structure prediction server (PS)^2^-V2 and this is the automated web server by the MODELLER tool. The model contains the highest sequence alignment (99.24%) and the identity was 63.36%. The structure resembled the continuous beta parallel strands (TIM Barrel) and alpha helices with inter connecting loops (Figure 7a). As earlier reports showed that the lipocalin members share structural similarity with functional motifs [19]. Then, the furnished EULP model was analyzed for identificaiton of binding site using the Q-SiteFinder tool, in which the model depicted the ten different binding sites (Figure 7b). Further, the EULP model was subjected for structure validation using phi/psi value obtained by Ramachandran plot present in the PROCHEK tool from the SAVeS server. Validation of EULP showed that the residues in most favoured regions got nearer 95.7%, and residues in additional allowed regions close to 4.3%. The best model would be anticipated to have over 90% in the most favoured regions and, thus, in EULP having more than this favoured regions (Figure 8), gets validated as belonging to lipocalin superfamily protein.

Hurst et al. [48] furnished a detailed account about improvement of longivity of the pheromonal compounds in the environment through the urine scent marking of male mouse using lipocalin as the binding protein. We reported the compounds 1-chlorodecane, a predominant urinary volatile [12], and farnesol of preputial gland [5] bound with 17 kDa and 21 kDa lipocalin protein, respectively, in male commensal rat. Aphrodisin, a lipocalin present in hasmster vaginal secretion, has been shown to serve a dual role of (a) by itself functiong as a pheromone, and (b) binding with pheromonal compound(s), thereby influencing the pheromonal effects [49]. Since these earlier studies provide elaborate information about bound form of lipocalin, and also lipocalins by themselves are capable of acting as pheromones, the 14.5 kDa lipocalin protein substantiated so in the sequence similarity and the structural features, identified in the urine of female commensal rat may have functional significance in sexual communication. Importantly, its high levels of prevalence during estrus would reveal the readiness of the female to entertain the male by way of mating behavior and coitus.

### Conclusions

In this study, we have reported for the first time the presence of lipocalin family protein in urine of a female rodent. The expression of this protein in the urine has a correlation with the phases in the estrous cycle, this being the highest during estrus and metestrus. That the protein we dealt with here is a lipocalin is established from homology similarity, sharing β-parallel structures with linked α-helices and most reported -GXW- motif region. We suggest that the 14.5 kDa lipocalin family rodent urinary protein might be involved as the shuttle for chemosignal communication, probably inviting the counterpart. The present finding will have far reaching implications in pheromone biology, such as search for female urinary lipocalin family protein in other rodents and other mammals as well and application in pheromone trap for rodents.

## Supporting Information

Figure S1 **Vaginal cytology using SEM analysis.** The vaginal secretion was smeared and observed the presence of cells through SEM. Estrus (cornified epithelial cell) and Diestrus (leucocytes cell), the arrow indicates the respective cells.
(TIF)

Figure S2 **SDS-PAGE of commensal rat urinary protein.** The figures indicate the estrous cycle urinary protein expression of three individual female rat over two cycles. (PE) Proestrus, (E) Estrus, (ME) Metestrus, (DE) Diestrus. A-1, 2 (Animal 1, cycle 1 and 2), B-1, 2 (Animal 2, cycle 1 and 2), C-1, 2 (Animal 3, cycle 1 and 2). Note: Animal 1, cycle 2 gel image is given as representative gel in the mauscript.
(TIF)

Table S1 **EULP functional motif sites.** Functional motif site of EULP was predicted by DIAL server.
(DOCX)
