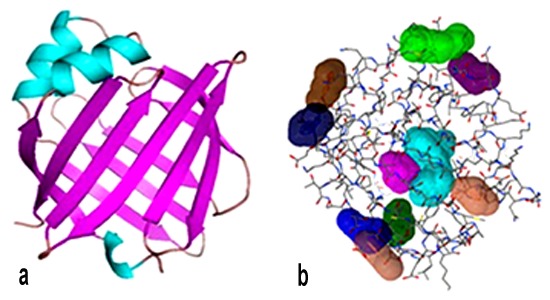# Correction: Urinary Lipocalin Protein in a Female Rodent with Correlation to Phases in the Estrous Cycle: An Experimental Study Accompanied by *In Silico* Analysis

**DOI:** 10.1371/annotation/8304f125-843b-40b3-9a48-9a86a713fde4

**Published:** 2013-09-10

**Authors:** Subramanian Muthukumar, Durairaj Rajesh, Ganesan Saibaba, Alagersamy Alagesan, Rengasamy Lakhsminarayanan Rengarajan, Govindaraju Archunan

The labels "A" and "B" did not exist in Figure 7. A correct version of the figure is available here: 

**Figure:**