# Supplementary figures and images for: Urinary Lipocalin Protein in a Female Rodent with Correlation to Phases in the Estrous Cycle: An Experimental Study Accompanied by In Silico Analysis
Source: PLoS One. 2013 Aug 14;8(8):e71357. doi: 10.1371/journal.pone.0071357 (PMC3743767; doi:10.1371/journal.pone.0071357)

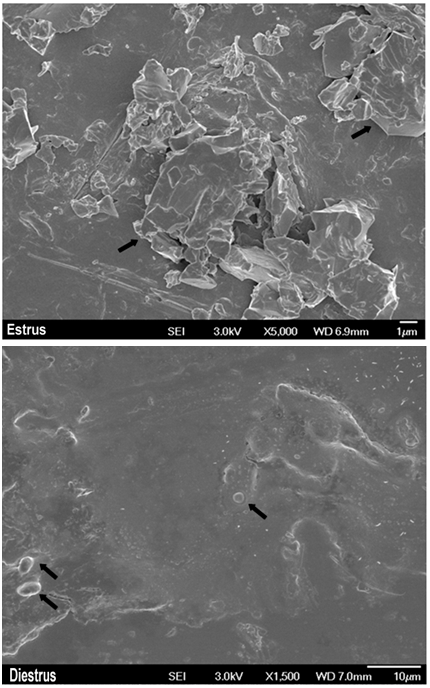

Supplement: Figure S1 — Vaginal cytology using SEM analysis. The vaginal secretion was smeared and observed the presence of cells through SEM. Estrus (cornified epithelial cell) and Diestrus (leucocytes cell), the arrow indicates the respective cells. (TIF) [file pone.0071357.s001.tif]

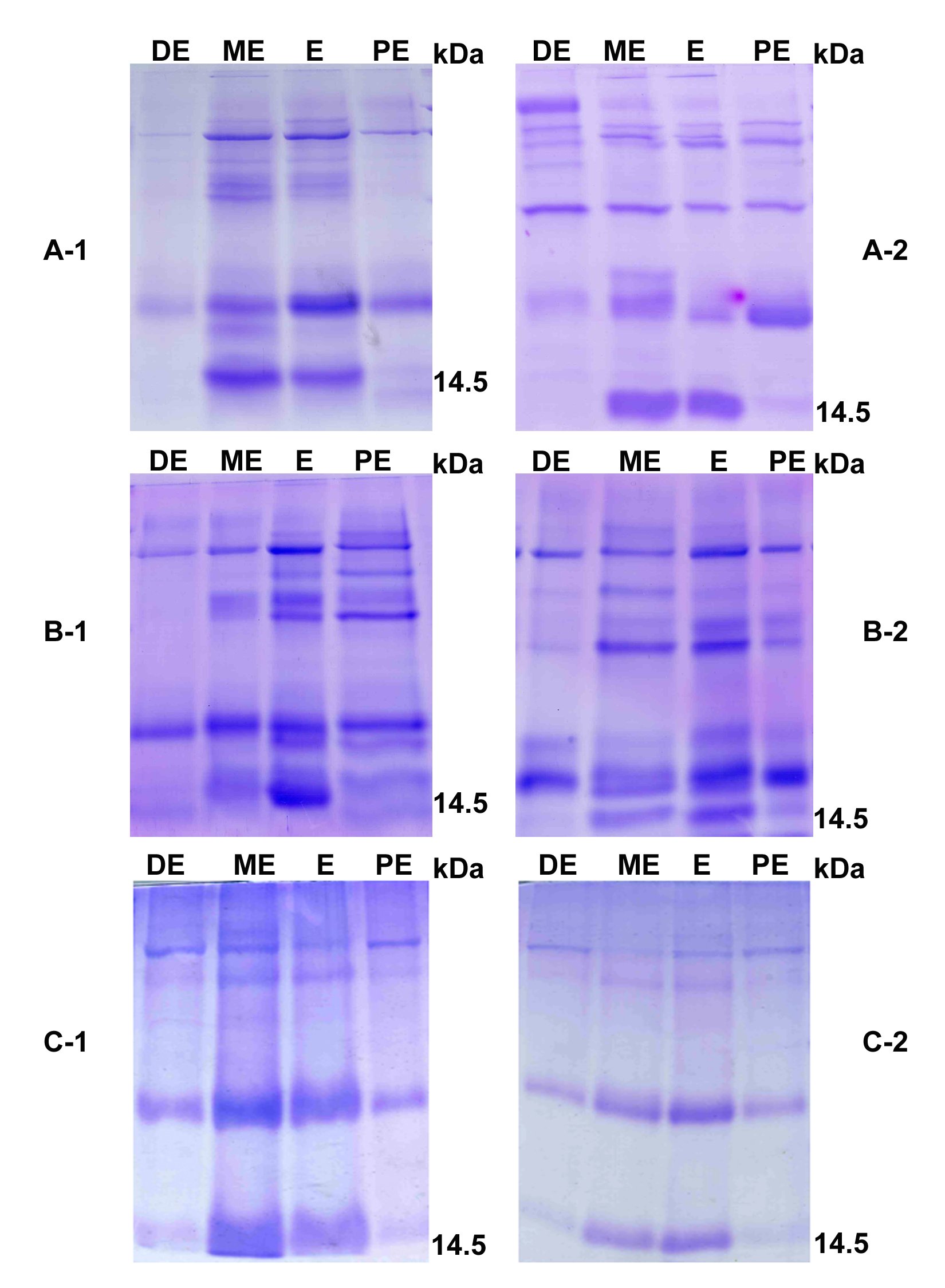

Supplement: Figure S2 — SDS-PAGE of commensal rat urinary protein. The figures indicate the estrous cycle urinary protein expression of three individual female rat over two cycles. (PE) Proestrus, (E) Estrus, (ME) Metestrus, (DE) Diestrus. A-1, 2 (Animal 1, cycle 1 and 2), B-1, 2 (Animal 2, cycle 1 and 2), C-1, 2 (Animal 3, cycle 1 and 2). Note: Animal 1, cycle 2 gel image is given as representative gel in the mauscript. (TIF) [file pone.0071357.s002.tif]
