# Supplementary material for: Urinary Lipocalin Protein in a Female Rodent with Correlation to Phases in the Estrous Cycle: An Experimental Study Accompanied by In Silico Analysis
Source: PLoS One. 2013 Aug 14;8(8):e71357. doi: 10.1371/journal.pone.0071357 (PMC3743767; doi:10.1371/journal.pone.0071357)
Supplement: Table S1 — EULP functional motif sites. Functional motif site of EULP was predicted by DIAL server. (DOCX) [file pone.0071357.s003.docx]

**Table S1. EULP functional motif sites**

| **Functional Motifs** | | |
| --- | --- | --- |
| **Start residue** | **End residue** | **Motif** |
| **N-glycosylation site** | | |
| **101** | 104 | NETT |
| **Tyrosine sulfation site** | | |
| **13** | 27 | ssenfdeYmkalgvg |
| **Protein kinase C phosphorylation site** | | |
| **2** | 4 | SnK |
| **8** | 10 | TwK |
| **30** | 32 | TrK |
| **44** | 46 | SkK |
| **51** | 53 | TiR |
| **64** | 66 | SfK |
| **104** | 106 | TiK |
| **Casein kinase II phosphorylation site** | | |
| **74** | 77 | TtaD |
| **N-myristoylation site** | | |
| **25** | 30 | GVglAT |
| **100** | 105 | GNetTI |
| **Cytosolic fatty-acid binding proteins signature** | | |
| **7** | 24 | GtWkLvsseNFDeYMkaL |

Functional motif site of EULP was predicted by DIAL server.
